# Machine learning approaches for predicting the structural number of flexible pavements based on subgrade soil properties

**DOI:** 10.1038/s41598-025-13852-0

**Published:** 2025-08-06

**Authors:** Asadullah Ziar

**Affiliations:** Department of Civil Engineering, Ghazni Technical University, Ghazni, 2301 Afghanistan

**Keywords:** Machine learning, Structural number, Flexible pavement, Subgrade soil properties, Bisection method, Civil engineering, Computer science

## Abstract

This study presents a machine learning approach to predict the structural number of flexible pavements using subgrade soil properties and environmental conditions. Four algorithms were evaluated, including random forest, extreme gradient boosting, gradient boosting, and K nearest neighbors. The dataset was prepared by converting resilient modulus values into structural numbers using the bisection method applied to the American Association of State Highway and Transportation Officials 1993 design equation. Input variables included moisture content, dry unit weight, weighted plasticity index, and the number of freeze and thaw cycles. Each model was trained and tested using standard performance metrics. Gradient boosting achieved the highest accuracy with a determination coefficient of 0.917. Moisture content was identified as the most significant predictor in most models. The findings demonstrate that machine learning models can accurately predict pavement thickness requirements based on readily available soil and environmental data. This approach reduces reliance on expensive and time-consuming laboratory tests and provides a practical and efficient tool for pavement design. This study highlights the potential of machine learning models in enhancing pavement design by accurately predicting structural performance parameters based on soil and environmental factors.

## Introduction

The American Association of State Highway and Transportation Officials (AASHTO) design method for flexible pavements is one of the most widely adopted approaches, both within the United States and internationally. The AASHTO Guide for the Design of Pavement Structures (AASHTO) 1993 integrates a comprehensive range of parameters, including traffic loading, material properties, drainage conditions, environmental factors, reliability, prediction variability, and performance trends. The traditional AASHTO pavement design methodology employs these parameters to estimate the required structural capacity of the pavement, expressed through an index known as the structural number (SN). This index quantifies the overall structural strength needed and is then translated into the corresponding thicknesses of pavement layers, based on the relative structural contributions of the materials used, as represented by their respective layer coefficients^1^. The fundamental design equation for flexible pavements is presented in Eq. (1).

1 $$\:{\text{log}}_{\text{10}}\text{(}{\text{W}}_{\text{18}}\text{)}\text{=}{{\text{Z}}_{\text{R}}\text{S}}_{\text{o}}\text{+\:9.36}{\text{log}}_{\text{10}}\text{(}\text{SN+1)\:-\:0.20\:+}\frac{{\text{log}}_{\text{10}}\left[\frac{\text{ΔPSI}}{\text{4.2\:\--\:ΔPSI}}\right]}{\text{0.40\:+\:}\frac{\text{1094}}{{\left(\text{SN+1}\right)}^{\text{5.19}}}}\text{\:+\:2.32}{\text{log}}_{\text{10}}\left(\text{MR}\right)\text{-8.07}$$

In Eq. (1), W_18_ represents the predicted number of 18-kips (80 kN) equivalent single axle loads (ESALs) that the pavement structure is expected to carry over its design life. The term Z_R_ denotes the standard normal deviate corresponding to the desired reliability level, while S_o_​ is the combined standard deviation, accounting for variability in traffic prediction and performance. The variable SN refers to the overall structural capacity of the pavement system. The expression ΔPSI indicates the change in the present serviceability index, defined as the difference between the initial and terminal serviceability levels. Lastly, MR ​ is the resilient modulus of the subgrade soil^2^.

Following the determination of the SN from Eq. (1), Eq. (2) is applied to convert this value into the corresponding thicknesses of the individual pavement layers. The designer must appropriately configure the pavement structure, keeping in mind that Eq. (2) accommodates up to three structural layers, while Eq. (2) yields multiple feasible thickness combinations, any selected configuration must comply with the minimum thickness requirements for surface and base layers as recommended by AASHTO 1993 guide^2^. The final thicknesses of the layers are then determined sequentially through equations (2a-2c).

2 $$\:\text{SN=}\left({\text{a}}_{\text{1}}{\text{D}}_{\text{1}}\text{+}{\text{a}}_{\text{2}}{{\text{m}}_{\text{1}}\text{D}}_{\text{2}}\text{+}{\text{a}}_{\text{3}}{\text{m}}_{\text{2}}{\text{D}}_{\text{3}}\right)$$

2a $$\:{\text{D}}_{\text{1}}\text{=\:}\frac{{\text{SN}}_{\text{1}}}{{\text{a}}_{\text{1}}}$$

2b $$\:{\text{D}}_{\text{2}}\text{=\:}\frac{{\text{SN}}_{\text{2}}\text{-\:}{\text{a}}_{\text{1}}{\text{D}}_{\text{1}}}{{\text{a}}_{\text{2}}{\text{m}}_{\text{2}}}$$

2c $$\:{\text{D}}_{\text{3}}\text{=}\frac{\text{SN\:-\:}{\text{a}}_{\text{1}}{\text{D}}_{\text{1}}\text{\:-}{\text{\:a}}_{\text{2}}{\text{m}}_{\text{2}}{\text{D}}_{\text{2}}}{{\text{a}}_{\text{3}}{\text{m}}_{\text{3}}}$$

Where, a_1_​, a_2_, and a_3_ represent the layer coefficients for the asphalt, base, and subbase layers, respectively, reflecting the relative structural contributions of each material. The terms m_2_ and m_3_​ denote the drainage coefficients for the base and subbase layers, accounting for the impact of moisture conditions and drainage quality. The variables SN_1_​ and SN_2​_ are obtained from Eq. (1), where each is calculated based on the resilient modulus of the base and subbase materials, respectively. Meanwhile, D_1_​, D_2_​, and D_3_ correspond to the thicknesses (in inches) of the asphalt, base, and subbase layers, in the same order.

The SN, as defined in Eq. (2), represents the overall structural capacity of a pavement resting on subgrade soil. As noted by Karballaeezadeh et al. (2020), SN is a positive value that is typically highest at the beginning of the pavement’s service life. As traffic continues to pass over the pavement, its initial structural capacity gradually decreases due to progressive deterioration^3^.

At the time of flexible pavement design, the maximum structural number is calculated via the AASHTO design equation for a specific design traffic load, design parameters, and subgrade soil quality. The subgrade quality is commonly defined by its MR or California bearing ratio (CBR). However, conducting MR and CBR tests is often costly and time-consuming. Therefore, alternative techniques are needed to estimate the total SN of the pavement on the basis of basic subgrade soil properties and environmental factors, such as freeze‒thaw cycles.

Numerous studies conducted by previous researchers have focused on the prediction, evaluation, estimation, and determination of pavement structural capacity during its service life, commonly referred to as the effective structural number (SN_eff_). These studies are widely attributed to various scholars who have made significant contributions to the assessment and modeling of pavement performance^3–7^.

Among the studies mentioned above, Karballaeezadeh et al.^3^presented a study titled *“*Estimation of flexible pavement structural capacity using machine learning techniques*”*. In their work, the authors proposed a machine learning-based approach for estimating the effective structural number (SN_eff_), which represents pavement strength during evaluation. They used three algorithms, including gaussian process regression (GPR), M5P model tree (M5P), and random forest. The model inputs were surface deflections and surface temperature, allowing the estimation of SN_eff_ without the use of ground penetrating radar, thereby reducing testing cost and complexity. Their dataset included 759 pavement sections from the Semnan and Khuzestan provinces in Iran. The model performance was evaluated using performance metrics such as coefficient of determination (R^2^, root mean square error (RMSE) and mean absolute error (MAE), with random forest showing the highest accuracy.

Another relevant study by Abd El-Raof et al. (2020)^4^titled *“*Structural number prediction for flexible pavements using the long-term pavement performance data*”*, focused on improving existing models for predicting SN_eff_ using falling weight deflectometer (FWD) data from the long-term pavement performance (LTPP) database. A key limitation in earlier models was the lack of temperature correction, which affects both the modulus of the asphalt concrete layer and FWD deflections. This study applied temperature correction to standardize the asphalt modulus and FWD peak deflection at 21 °C. The analysis used 1293 FWD test points from 14 pavement test sections. The improved models showed greater accuracy, and less bias compared to the original formulations, and their effectiveness was confirmed through validation with additional LTPP data.

Although these studies are related in terms of predicting structural numbers, their objectives differ from ours. While their focus is on predicting SN_eff_ during pavement evaluation, our study aims to predict the initial SN at the design stage, before any degradation occurs. This distinction is important because our approach forecasts pavement capacity based on subgrade soil properties and environmental conditions available at the design time. To data, no previous research has predicted the initial SN at the design stage, which is essential for determining pavement layer thickness. To address this research gap and provide a foundation for future studies in transportation engineering, the primary objective of this study is to develop and evaluate four machine learning algorithms, namely random forest, extreme gradient boosting, gradient boosting, and K nearest neighbors, which are abbreviated throughout the article as RFR, XGBR, GBR, and KNR respectively, for the accurate prediction of the total SN of flexible pavements.

This research proposes a novel data-driven approach that eliminates the need for time-consuming and costly MR and CBR testing. Instead, the model estimates SN via easily obtainable fundamental subgrade soil properties, including dry unit weight (γ_d_), moisture content (w in %), and the weighted plasticity index (wPI). Additionally, the number of freeze–thaw cycles (NFT) is incorporated as an environmental parameter. This approach not only enhances the efficiency and cost-effectiveness of pavement design but also improves the prediction accuracy through the integration of machine learning techniques. Ultimately, the proposed framework aims to support future advancements in the design of both flexible and rigid pavements by enabling data-driven, reliable, and practical engineering solutions.

## Data description and preprocessing for machine learning models

The dataset used in this study was originally compiled and shared by^8^ and is publicly available as supplementary material accompanying their article. It includes experimental results of MR tests conducted on compacted subgrade soils classified under both the AASHTO system (A-4, A-6, and A-7-6) and the Unified Soil Classification System (USCS) (CL, CH, and CL-ML). The dataset was accessed and downloaded from the supplementary materials provided by the publisher. The compiled data were originally sourced from multiple studies, including^9–12^.

The key input variables influencing the MR of the subgrade soils include the weighted plasticity index (wPI), dry unit weight (γ_d_, in kN/m^3^), confining stress (σ_c_, in kPa), deviator stress (σ_d_, in kPa), number of freeze–thaw cycles (NFT), and moisture content (w, in %). Zou et al. (2021)^8^ utilized this dataset to develop gene expression programming (GEP) and artificial neural network (ANN) models for predicting the resilient modulus of pavement subgrade soils based on their physical properties, loading conditions, and environmental influences.

In the present study, rather than directly using the dataset for MR prediction, it was revised and adapted to meet the specific objective of this research, which was to develop and evaluate four machine learning algorithms, namely RFR, XGBR, GBR, and KNR, for predicting the total structural number (SN) of flexible pavements. The prediction was based on subgrade soil properties and environmental conditions under a defined traffic level and predetermined pavement design parameters. To achieve this, the dataset was transformed by estimating SN values from MR values using the bisection method. This method involves solving the AASHTO 1993 empirical pavement design equation. Equation (1) defines the relationship between SN and design variables such as the predicted traffic loading (W18), the standard normal deviate for the reliability level (ZR), the overall standard deviation (So), the serviceability loss (ΔPSI), and the subgrade resilient modulus (MR). The adopted design parameters were selected in accordance with the AASHTO 1993 Guide for Design of Pavement Structures and include a reliability (R) of 95%, ZR of -1.282, So of 0.45, and ΔPSI of 2.5. The overall standard deviation, So, accounts for the combined variability in traffic loading, material properties, and construction quality, reflecting the inherent uncertainties in pavement design. The value of So = 0.45 used here aligns with the AASHTO recommended range for flexible pavements, providing a conservative yet realistic basis for the analysis^2^.

A cumulative traffic loading of W18 = 5 million ESALs was selected to represent typical medium to high volume roads. The reliability level (R) was set to 95%, which falls within the recommended range of 85–99.9% for urban freeways and principal arterials (see Table 1), ensuring the design accounts for variability in traffic loads, material properties, and construction quality. While the AASHTO 1993 guide^2^ suggests a typical initial serviceability index (Pi) of 4.2 and a terminal serviceability index (Pt) of 2.5 (yielding a standard ΔPSI of 1.7) this study adopts a more conservative ΔPSI of 2.5. This decision reflects a higher-initial performance expectation and a stricter minimum serviceability threshold before rehabilitation, consistent with practices applied in critical or high priority roadway systems. These parameter choices align with the AASHTO design framework while supporting a robust and conservative pavement structure analysis. The parametric study of these parameters is presented in Sect. 4.1.

In Eq. (1), W₁₈, Z_R_, ΔPSI, S_o,_ and MR are known parameters, whereas SN is the unknown variable to be determined. To compute SN, an objective function was defined as the absolute difference between the left-hand side (log₁₀(W₁₈)) and the right-hand side of the equation. The bisection method, implemented in Python, was employed to find the root of this objective function by iteratively refining the SN value until convergence was achieved within a predefined tolerance (e.g., 0.001). This root-finding procedure was repeated for each MR value in the dataset to generate corresponding SN values. As a result, a new dataset was created for this study, which includes weighted plasticity index (wPI), dry unit weight (γ_d_, in kN/m^3^), freeze–thaw cycles (NFT), and moisture content (w, in %). as input variables, with SN as the output. These variables directly influence the MR, which governs SN via the AASHTO design equation. Higher moisture content reduces MR by weakening soil structure^13^while lower dry unit weight indicates poor compaction and lower stiffness. An increase in plasticity index tends to decrease MR by increasing soil deformability, particularly under soaked conditions^14^. Additionally, freeze–thaw cycles degrade MR by breaking down the soil structure, thereby increasing pavement strain and the required SN^15^. Collectively, these variables capture essential factors affecting pavement performance. A summary of their statistical characteristics is presented in Table 2.

In the dataset, the number of freeze–thaw cycles (NFT) represent laboratory-simulated environmental conditioning, not field-observed values. Different soil specimens were compacted and subjected to either closed or open system freeze–thaw cycles under varying temperature ranges and durations, depending on soil type, as documented in the original study^8^. For example, some specimens were tested using closed system freeze-thaw cycles, where moisture content remained constant, and each cycle included freezing at − 20 °C for 12 h followed by thawing at 20 °C for 12 h. Other soils underwent closed system cycles at − 5 °C for 16 h and thawing at 30 °C for 8 h. In contrast, certain groups were subjected to open system freeze-thaw cycles, where free water was available during conditioning, and specimens were frozen for 24 h at temperatures not higher than − 23 °C and thawed for 23 h at 21 °C. Another set used a 12-hour freeze at − 15 °C followed by 12-hour thawing at approximately 23 °C. These controlled variations represent a range of realistic field conditions experienced in cold regions and simulate long term environmental effects such as strength loss and stiffness degradation. These conditioned samples were later used to measure MR through dynamic triaxial tests.

The freeze–thaw cycle is a physical process in which water within the soil repeatedly freezes and thaws as temperatures fluctuate above and below freezing. This phase change between ice and liquid water can significantly alter and degrade the physical properties of the soil^16,17^. Particular attention should be given to the design of flexible pavements in regions where the subgrade experiences freezing conditions, especially within the frozen pore zones. The wetting–drying and freeze–thaw cycles degrade the resilient modulus and strength of pavement materials, leading to increased tensile strain in the surface layer under traffic loading. As this strain grows, the risk of developing reticular (net-like) fatigue cracks rises, compromising the pavement’s structural integrity and long-term performance^8,15,18^.

**Fig. 1:**
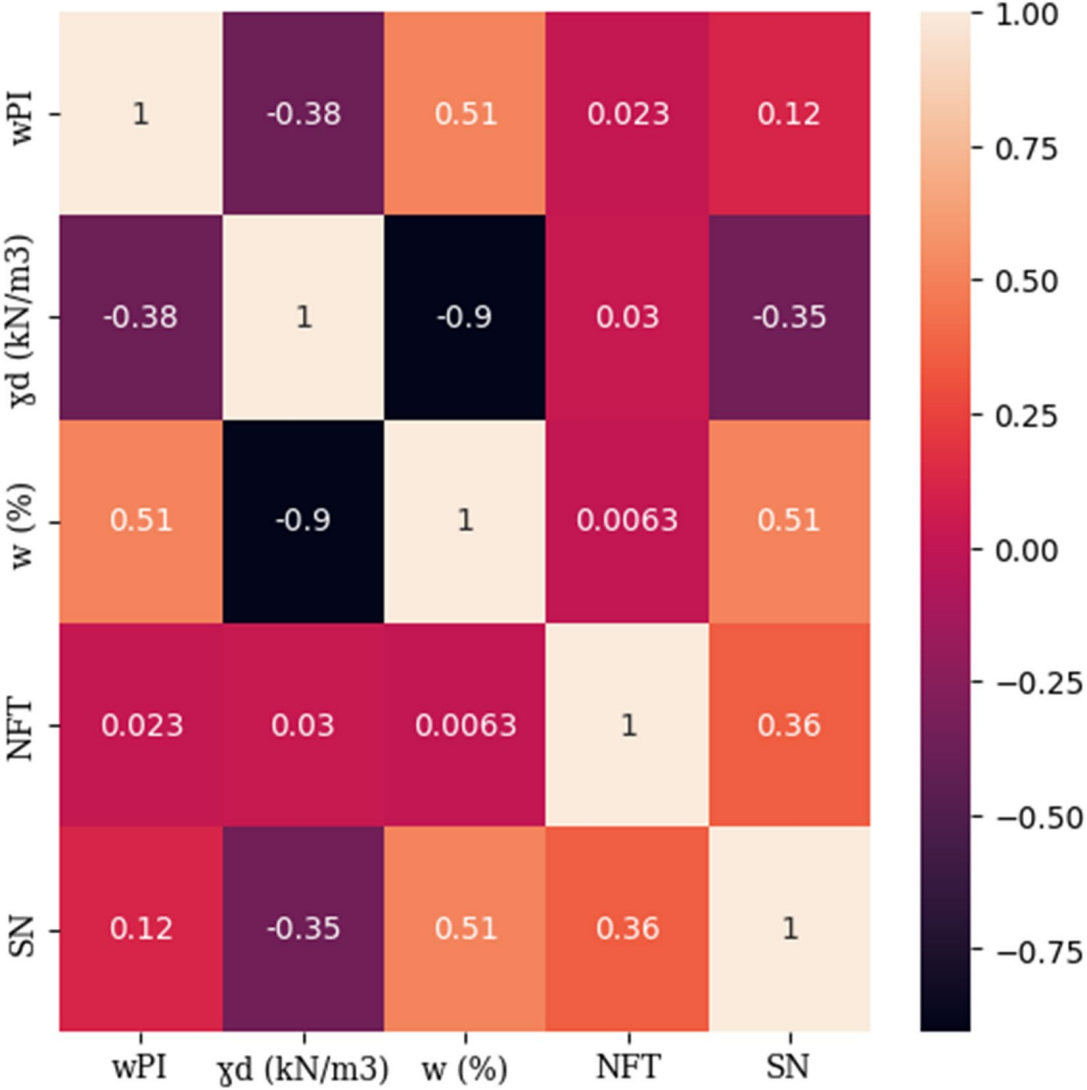
Correlation heatmap illustrates the linear relationships between input variables and the SN of flexible pavements. Light and dark colors indicate positive and negative correlations, respectively. Key variables include wPI, γ_d_, w, NFT, and SN.

The relationships between the input variables and the SN of flexible pavements are presented in Fig. 1 as a correlation heatmap based on Pearson correlation coefficients, which range from − 1 to + 1. These coefficients quantify the linear association between each pair of variables. In the heatmap, lighter colors represent stronger positive correlations, while darker colors, particularly purple to black tones, indicate stronger negative correlations. Among the input variables, moisture content (w) demonstrates the strongest positive correlation with SN (*r* = 0.51), suggesting that higher moisture levels are associated with increased pavement thickness requirements. This is followed by the NFT, which also shows a positive correlation with SN (*r* = 0.36), indicating that repeated environmental conditioning increases structural demand. In contrast, γ_d_ shows a moderate negative correlation with SN (*r* = − 0.35) and a strong inverse relationship with moisture content (*r* = − 0.90), highlighting their interdependence. Soils with lower dry density typically retain more moisture, thereby influencing pavement design. The weakest correlation is observed between the wPI and SN (*r* = 0.12), indicating a relatively minor direct effect. Although wPI is widely used in subgrade classification, its low correlation with SN is consistent with previous findings such as those by^19^who reported a weak negative relationship (*r* = − 0.025) between wPI and the MR. Since MR and consequently SN is more directly influenced by moisture content, dry unit weight, and freeze–thaw cycles, the effect of wPI is primarily expressed through its interaction with these variables, especially moisture content. This suggests that the predictive power of wPI is context dependent and more effectively captured through multivariate or interaction-based modelling approaches.

**Fig. 2:**
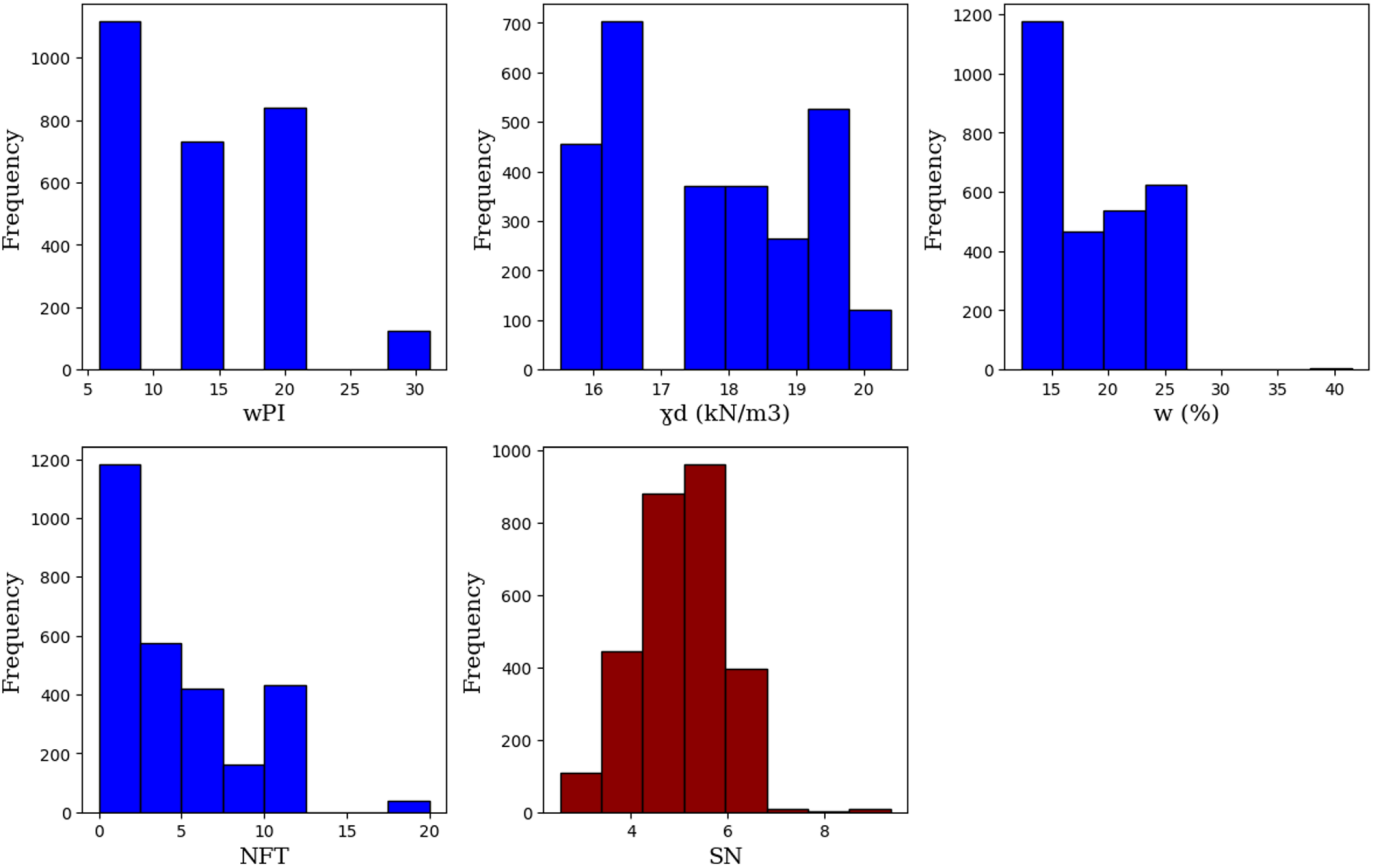
Histograms show the frequency distributions of each variable in the dataset.

Figure 2 displays the histograms of wPI, γ_d_, w, NFT, and SN, and each histogram displays the frequency of observations across value ranges, highlighting the spread, central tendency, and variability of the data. These visualizations provide a clear overview of the statistical characteristics of the dataset and may indicate the presence of skewness, clusters, or outliers.

**Table 1:** Recommended reliability levels (in %) for different functional classifications^2^.

Functional Classification	Urban	Rural
Interstate and other Freeways	85-99.9	80-99.9
Principal Arterials	80–99	75–95
Collectors	80–95	75–95
Local	50–80	50–80

**Table 2:** Statistical characteristics of input and output variables.

Parameters	wPI	γ_d_ (kN/m^3^)	w (%)	NFT	SN
Maximum	31.08	20.40	41.54	20.00	9.37
Minimum	5.82	15.50	12.30	0.00	2.53
Range	25.26	4.90	29.24	20.00	6.84
Mean	13.88	17.73	18.36	4.14	5.03
Median	13.16	17.77	17.30	3.00	5.08
Standard deviation	6.44	1.56	4.52	3.93	0.90

## Methodology

The primary objective of this study is to develop and evaluate four machine learning algorithms namely random forest, extreme gradient boosting, gradient boosting, and K nearest neighbors for predicting the total structural number of flexible pavements. This research also aims to support future studies in integrating these and other machine learning models into the design of both flexible and rigid highway pavements.

To achieve this, the structural number of flexible pavements was predicted using fundamental subgrade soil properties, including dry unit weight, moisture content, and the weighted plasticity index, which is defined as the product of the percentage passing through the No. 200 sieve and the plasticity index^20^. Additionally, the number of freeze–thaw cycles was included as an environmental factor. These input features were used across four prediction models, as detailed in the dataset description and preprocessing sections.

The dataset used for this study consists of a total of 2810 data points. It was pre-processed and divided into training and testing subsets, with 80% (2248 data points) allocated for training and 20% (562 data points) reserved for testing. Feature scaling was applied where necessary to ensure uniformity among the variables. Each model, namely RFR, XGBR, GBR, and KNR, was trained on the training set and evaluated on the test set using standard performance evaluation metrics including the R^2^RMSE, and MAE.

**Fig. 3:**
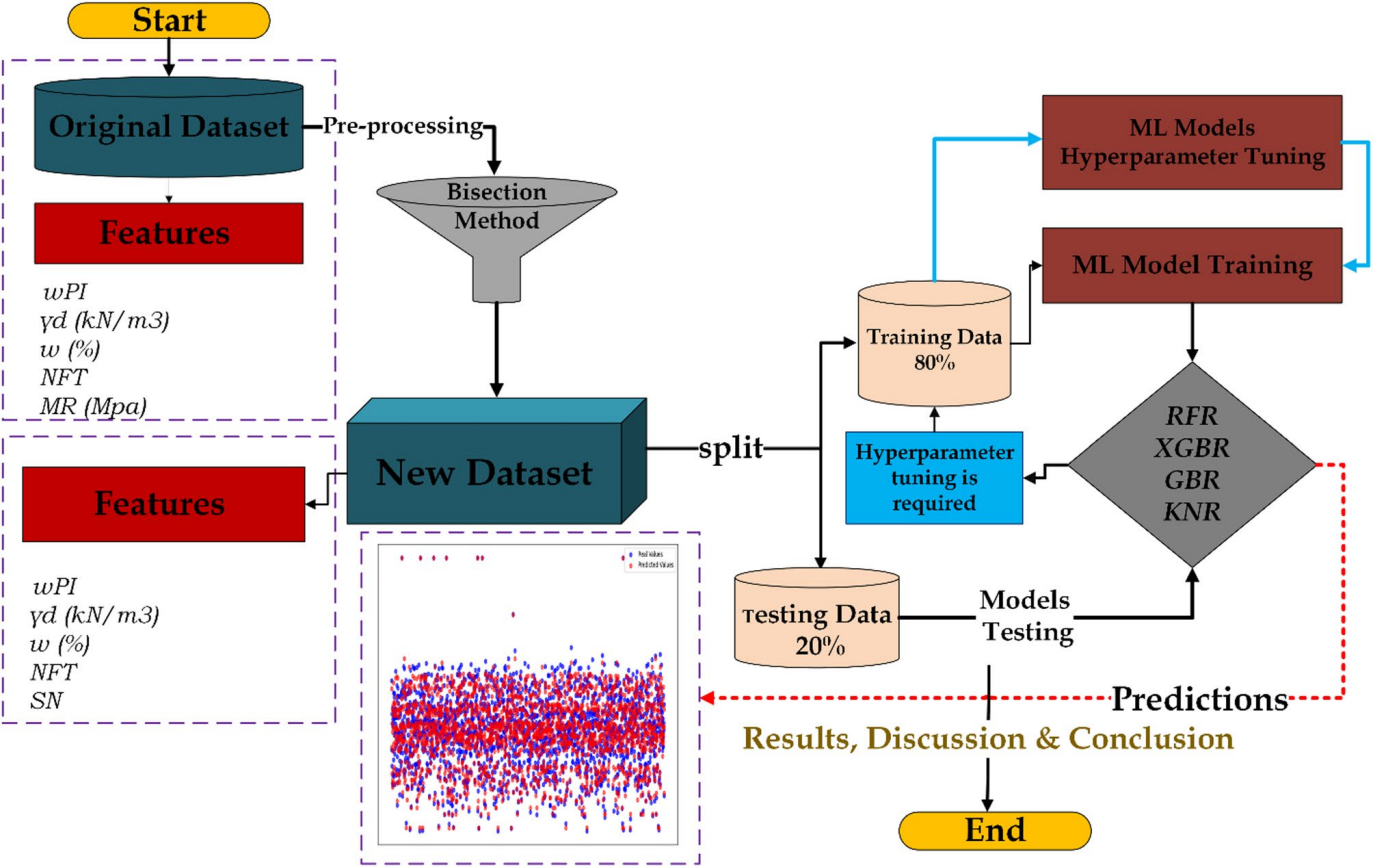
Schematic representation of the research methodology.

After evaluating the initial performance of the models, hyperparameter tuning was performed. The models were then retrained on the training set using the optimized hyperparameters and evaluated again on the test set using the same metrics. These metrics allowed for a comparative assessment of each model’s accuracy and generalization ability in predicting the structural number of pavement structures. The complete workflow of the study is shown in Fig. 3. The hyperparameter tuning will be discussed later.

To assess the contribution of each input feature in predicting the structural number, feature importance analysis was conducted for all four models. For the tree-based ensemble models (RFR, XGBR and GBR), built in feature importance metrics were extracted, which are typically based on the reduction in node impurity or gain. Since KNR is a nonparametric, instance-based model and does not provide intrinsic feature importance, a model agnostic technique, permutation importance, was applied. This method involves shuffling each input feature in the test set multiple times (30 repetitions) and observing the resulting decrease in model performance. The features that caused greater performance degradation were deemed more important. The feature importance results for all the models were visualized in a grouped horizontal bar chart to enable a direct comparison of each feature’s relative influence, as illustrated in Fig. and discussed in the result and discussion section.

### A brief overview of the machine learning models applied in our study

#### Random forest

**Fig. 4:**
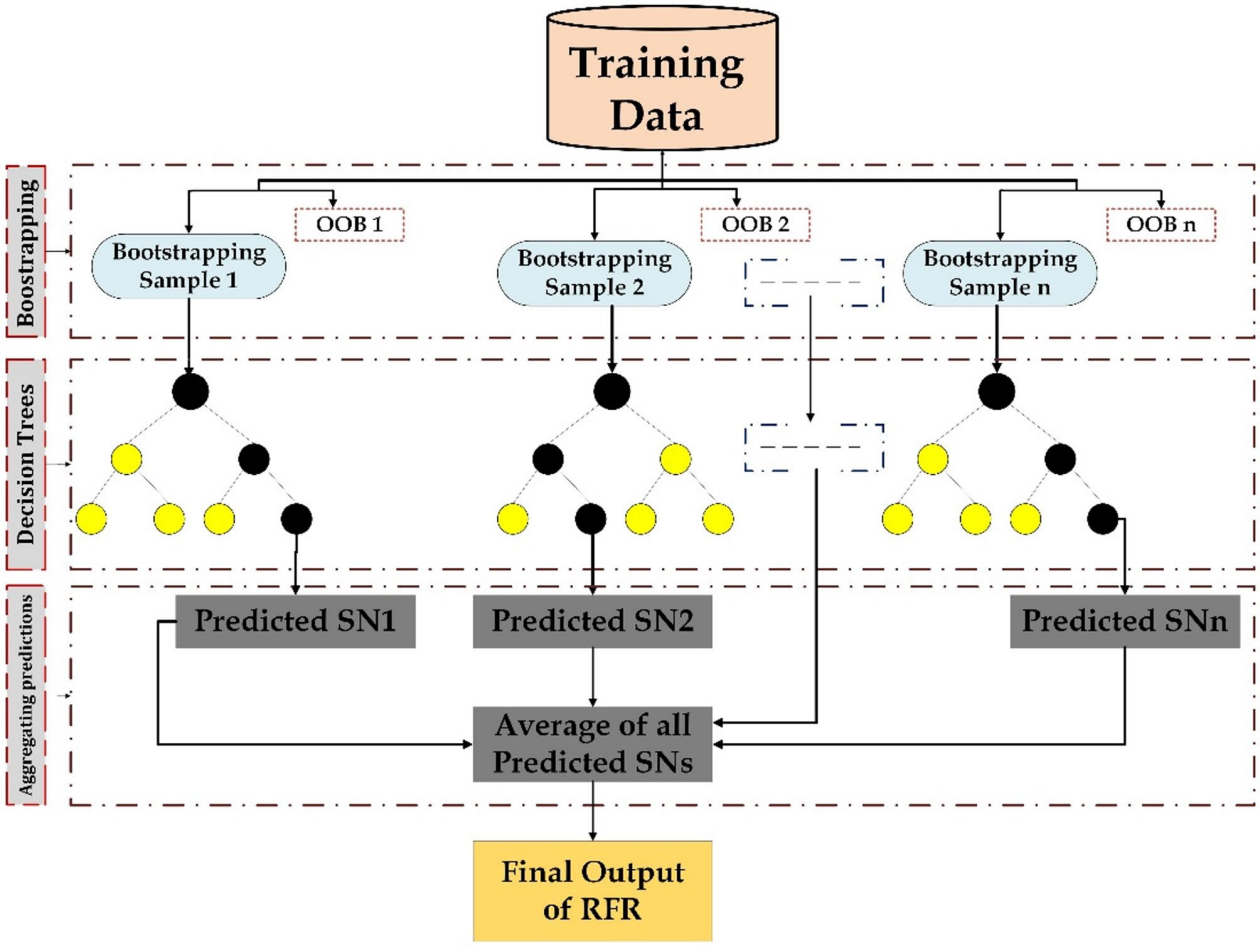
Schematic representation of the RFR model architecture.

RFR is an ensemble learning method that constructs multiple decision trees using bootstrapped samples of the dataset and random feature selection at each split^21^. The individual trees remain unpruned, and the final prediction is made by averaging the outputs of all trees for regression tasks or by majority voting in classification tasks. To increase diversity among the trees, RFR applies the bagging technique, in which each tree is trained on a randomly resampled subset of the original data with replacement. As a result, not all data points are used to train every tree. The samples that are excluded during the training of a specific tree, referred to as the k^th^ tree, are known as out of bag (OOB) samples^22^. These samples act as an internal validation set and enable the estimation of prediction error without requiring a separate dataset. At each split, the random forest selects the best feature from a randomly chosen subset rather than considering all features. This strategy reduces correlation between trees and enhances the generalization performance of the ensemble, even if it slightly lowers the performance of individual trees. The model layout of RFR is shown in Fig. 4.

#### Extreme gradient boosting

**Fig. 5:**
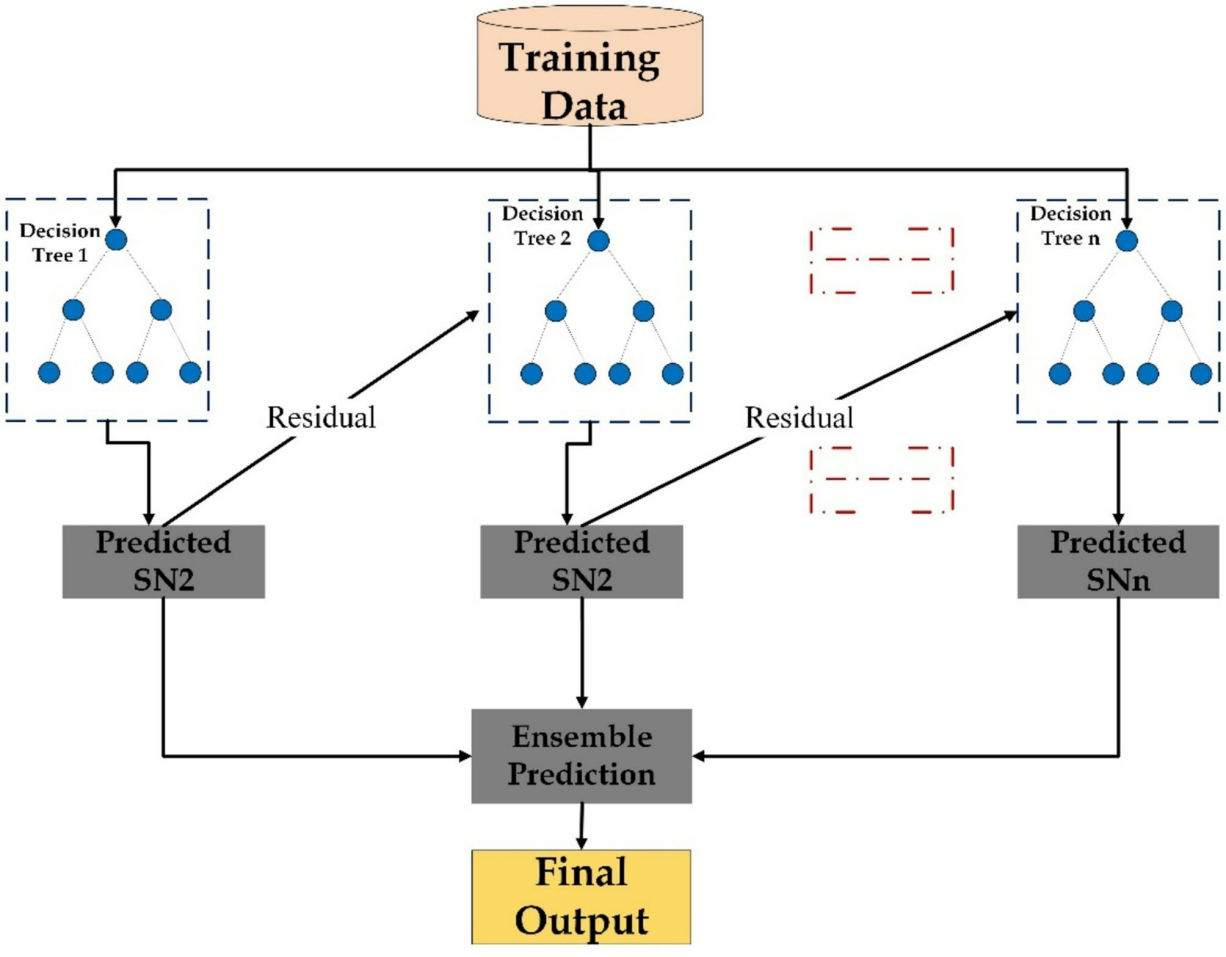
Schematic representation of the XGBR and GBR model architecture.

XGBR is a highly efficient and scalable machine learning algorithm known for delivering strong performance in various tasks including regression, classification, and ranking. Based on the gradient boosting framework, XGBR improves model accuracy by sequentially adding decision trees that correct the errors made by previous trees. In each iteration, a new tree is constructed to reduce the ensemble’s shortcomings by optimizing a defined loss function using gradient descent techniques. This process continues with each boosting step further refining the overall model until a stopping condition is met, such as reaching the maximum number of iterations or observing minimal improvement in error^23^. The schematic diagram of XGBR is shown in Fig. 5.

#### Gradient boosting

GBR is a powerful machine learning method widely applied in both research and practical settings. It is part of the boosting algorithm family and is well suited for addressing complex regression problems. GBR builds a strong predictive model by combining multiple weak learners, typically decision trees, in a sequential process. Each new model focuses on correcting the errors made by the previous one by using gradient descent to minimize a defined loss function. This iterative approach results in a highly accurate and dependable regression model^24^. The schematic diagram of GBR is shown in Fig. 5.

#### K-nearest neighbors

KNR is a nonparametric method that classifies data points based on the majority vote of their neighbors. It is simple and intuitive, making it effective for certain types of regression and classification problems^25^. Nearest neighbor methods rely on the labels of the K nearest data points in the feature space. As local learning techniques, they tend to perform well, particularly when working with large datasets and low-dimensional spaces. There are several variants of nearest neighbor algorithms designed to handle tasks such as multilabel classification, regression, and semi supervised learning, making them applicable to a wide range of machine learning problems. Moreover, decision theory offers valuable insights into the behavior and performance of nearest neighbor models, helping them to understand their strengths and limitations^26^.

### Model evaluation metrics

To evaluate the performance of the developed machine learning models, several statistical metrics were used. These include root mean square error, defined in Eq. (3), mean absolute error, presented in Eq. (4), and the coefficient of determination, shown in Eq. (5). Each of these metrics reflects a different aspect of model accuracy. As explained by Kumar and Pratap (2024) root mean square error measures the standard deviation of prediction errors and gives greater weight to larger errors. Mean absolute error represents the average absolute difference between predicted and actual values, providing a clear and interpretable measure of accuracy. The coefficient of determination indicates how well the model explains the variability in the observed data^27^. Together, these metrics form a strong basis for evaluating and comparing the predictive performance and reliability of the models.

3 $$\:\text{R}\text{M}\text{S}\text{E}=\frac{1}{\text{n}}\sqrt{{\sum\:}_{\text{i}=1}^{\text{n}}{\left({\text{y}}_{\text{Actual\_SN}}\:-{\:\text{y}}_{Predicted\_SN}\right)}^{2}}$$

4 $$\:\text{MSE=}\frac{1}{n}{\sum\:}_{\text{i=1}}^{n}{\left({\text{y}}_{\text{Actual\_SN}}\:-\:{\text{y}}_{Predicted\_SN}\right)}^{2}$$

5 $$\:\text{R}2\:=1-\:\frac{{\sum\:}_{\text{i}=1}^{\text{n}}{\left({\text{y}}_{\text{A}\text{c}\text{t}\text{u}\text{a}\text{l}\_\text{S}\text{N}}\:-\:{\text{y}}_{\text{p}\text{r}\text{e}\text{d}\text{i}\text{c}\text{t}\text{e}\text{d}\_\text{S}\text{N}}\right)}^{2}}{\sum\:_{\text{i}=1}^{\text{n}}{\left({\text{y}}_{Actual\_SN}\:-\:\stackrel{-}{\text{y}}\:\right)}^{2}}$$

In these formulas, n represents the number of samples, and y bar denotes the mean of the actual values in the dataset.

### Hyperparameter tuning of predictive models

Rather than relying on the default hyperparameters provided by the scikit-learn and xgboost libraries, hyperparameter optimization was employed to improve the performance and robustness of the machine learning models. This process which is a fundamental and widely adopted step in developing reliable and generalizable predictive models^28–30^was carried out using a grid search approach with 5-fold cross-validation to enhance predictive accuracy. The complete search space used for each model is summarized in Table 3.

For the RFR, the search space included variations in max_depth, n_estimators, max_features, and min_samples_leaf. The best performing configuration was max_depth = 10, max_features=’sqrt’, min_samples_leaf = 1, and n_estimators = 500, achieving a cross-validated R^2^ score of 0.906. On the test set, the RFR attained an R^2^ of 0.916 and an RMSE of 0.254. The XGBR was optimized over parameters such as max_depth, n_estimators, learning_rate, colsample_bytree, and subsample. The optimal configuration of max_depth = 5, n_estimators = 100, learning_rate = 0.1, colsample_bytree = 1.0, and subsample = 0.8 achieved a cross-validated R^2^ of 0.914 and yielded an R^2^ of 0.916 and RMSE of 0.254 on the test set. Similarly, the GBR achieved its best performance with n_estimators = 100, learning_rate = 0.1, max_depth = 5, min_samples_split = 5, min_samples_leaf = 1, and subsample = 1.0. It recorded a cross-validated R^2^ of 0.914 and an R^2^ of 0.917 with RMSE of 0.253 on the test data. Lastly, the KNR was fine-tuned over n_neighbors, weights, and p, with the optimal parameters being n_neighbors = 7, weights=’distance’, and *p* = 1 (Manhattan distance). This model achieved a cross-validated R^2^ of 0.878 and showed strong predictive capability on the test set with an R^2^ of 0.898 and RMSE of 0.280.

In total, 600 were performed for RFR, 1200 for XGBR, 1080 for GBR, and 120 for KNR. All models were trained using 5-fold cross-validation with parallel processing (n_jobs = -1) to reduce computational time.

**Fig. 6:**
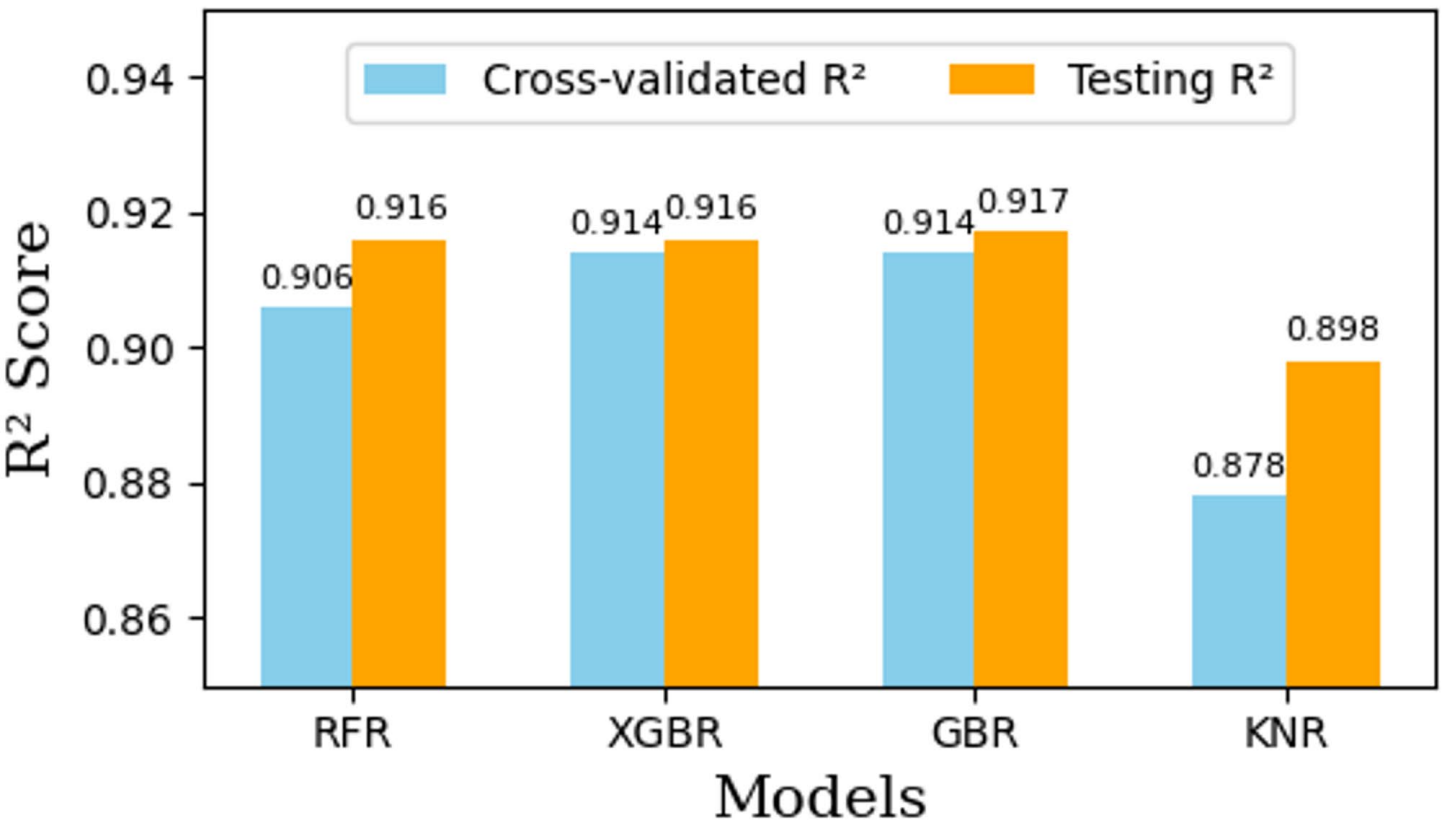
Comparison of Cross-validated and Testing R^2^ Scores for the developed models (RFR, XGBR, GBR, KNR). The close agreement between the two scores for each model indicates consistent performance and absence of overfitting.

Figure 6 illustrates the comparison between cross-validated R^2^ scores and testing R^2^ scores for the four developed machine learning models. As shown in the figure, the values of R^2^ on both the cross-validation and test datasets are closely aligned for all models. This close agreement indicates that the models exhibit consistent predictive performance and are not overfitted with the training data. The negligible differences between the cross-validated and test R^2^ scores suggest that the models generalize well to unseen data. This further confirms the effectiveness of the 5-fold cross-validation strategy employed during hyperparameter optimization, ensuring robust model evaluation and enhanced generalizability.

**Table 3:** Hyperparameter search space used for grid search.

Models	Hyperparameter	Search Space
RFR	n_estimators	100, 200, 300, 400, 500
	max_depth	3, 5, 7, 10
	max_features	sqrt, log2
	min_samples_leaf	1, 2, 4
XGBR	n_estimators	100, 200, 300, 400, 500
	max_depth	3, 5, 7, 10
	learning_rate	0.01, 0.05, 0.1
	subsample	0.8, 1.0
	colsample_bytree	0.8, 1.0
GBR	n_estimators	100, 200, 300
	learning_rate	0.05, 0.1, 0.2
	max_depth	3, 5, 7
	subsample	0.8, 1.0
	min_samples_split	2, 5
	min_samples_leaf	1, 2
KNR	n_neighbors	3, 5, 7, 9, 11, 15
	weights	uniform, distance
	P (Manhattan distance)	1, 2

## Results and discussion

**Fig. 7:**
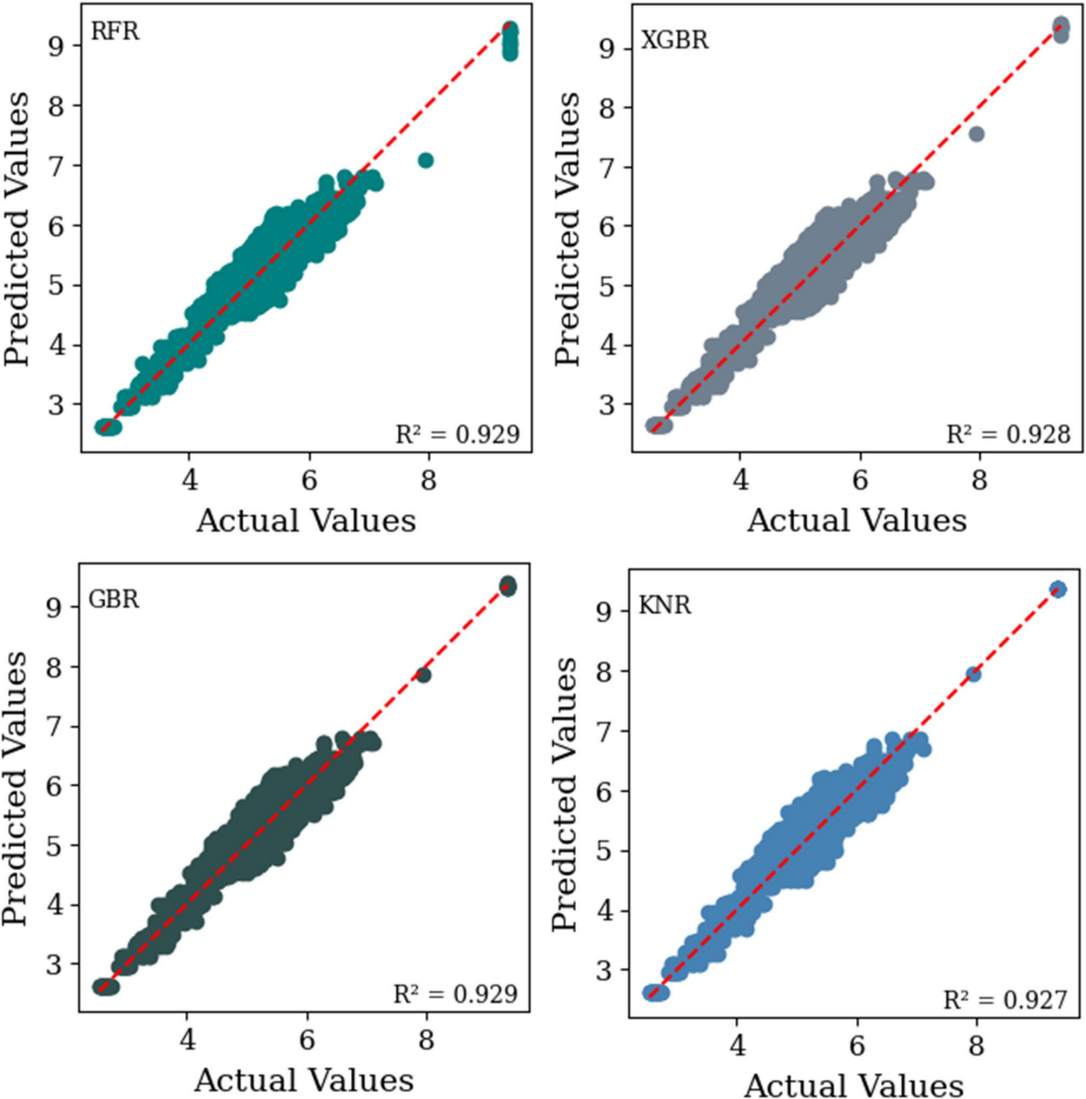
The scatter plot illustrates the relationship between the measured and predicted SN values for each machine learning model (RFR, XGBR, GBR, and KNR) in the training dataset.

**Fig. 8:**
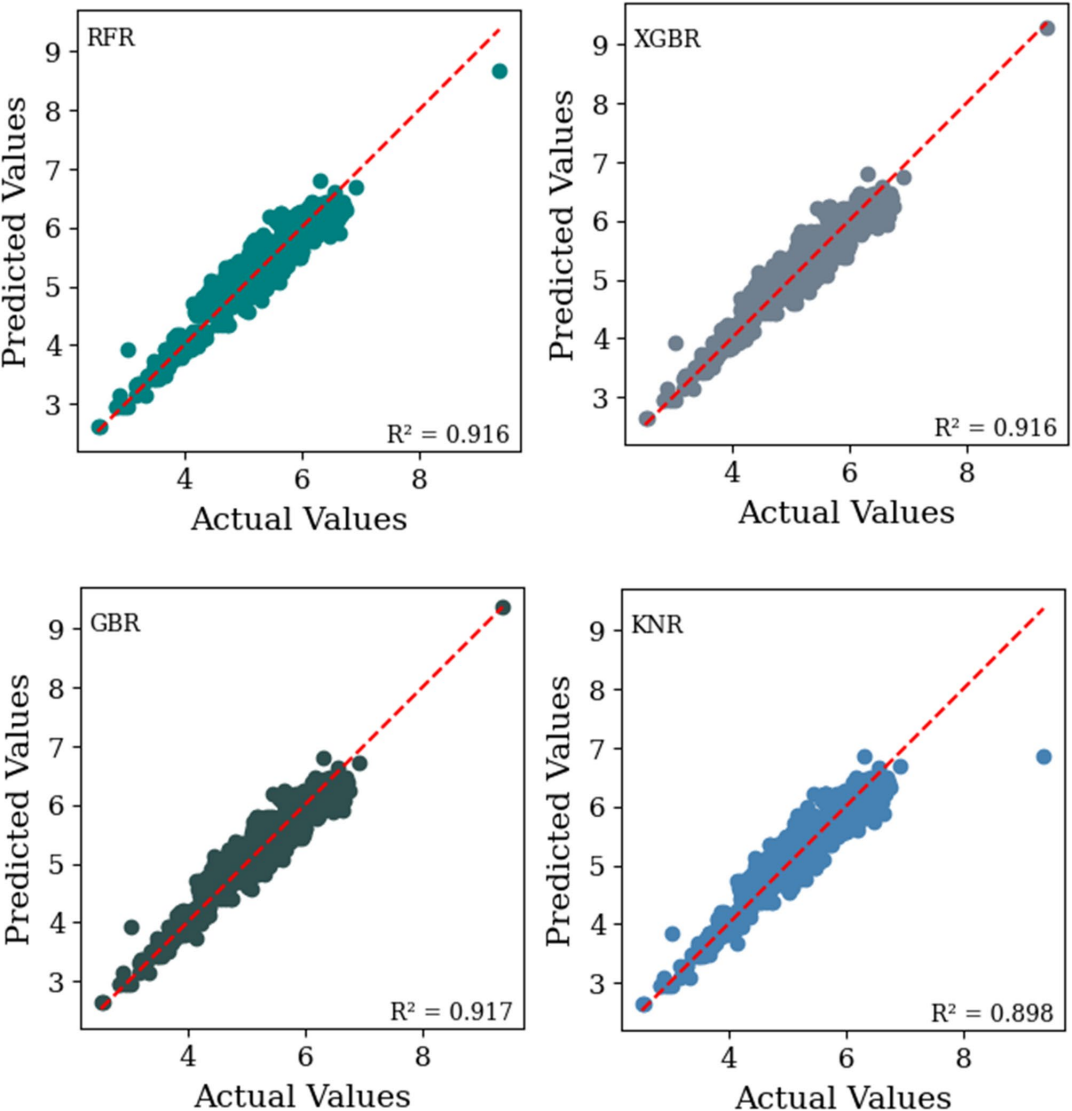
The scatter plot illustrates the relationship between the measured and predicted SN values for each machine learning model (RF, XGBR, GBR, and KNR) on the testing dataset.

This section presents the results of the developed machine learning models. Figure 7, along with Fig. 8, provides a comparative analysis of the predictive performance of four machine learning models namely RF, XGBR, GBR, and KNR, on the training and testing datasets. These models were employed to predict the total SN of flexible pavement based on the physical characteristics of subgrade soils and environmental factors for a specific traffic load, defined as 5 million repetitions of an 18-kip equivalent single-axle load (W18). The prediction was carried out using predefined design parameters in accordance with the AASHTO Guide for Design of Pavement Structures^2^. The selected parameters include reliability (R) = 90%, standard normal deviation for the reliability level (ZR) = -1.282, overall standard deviation (So) = 0.45, and serviceability loss (ΔPSI) = 2.5.

**Fig. 9:**
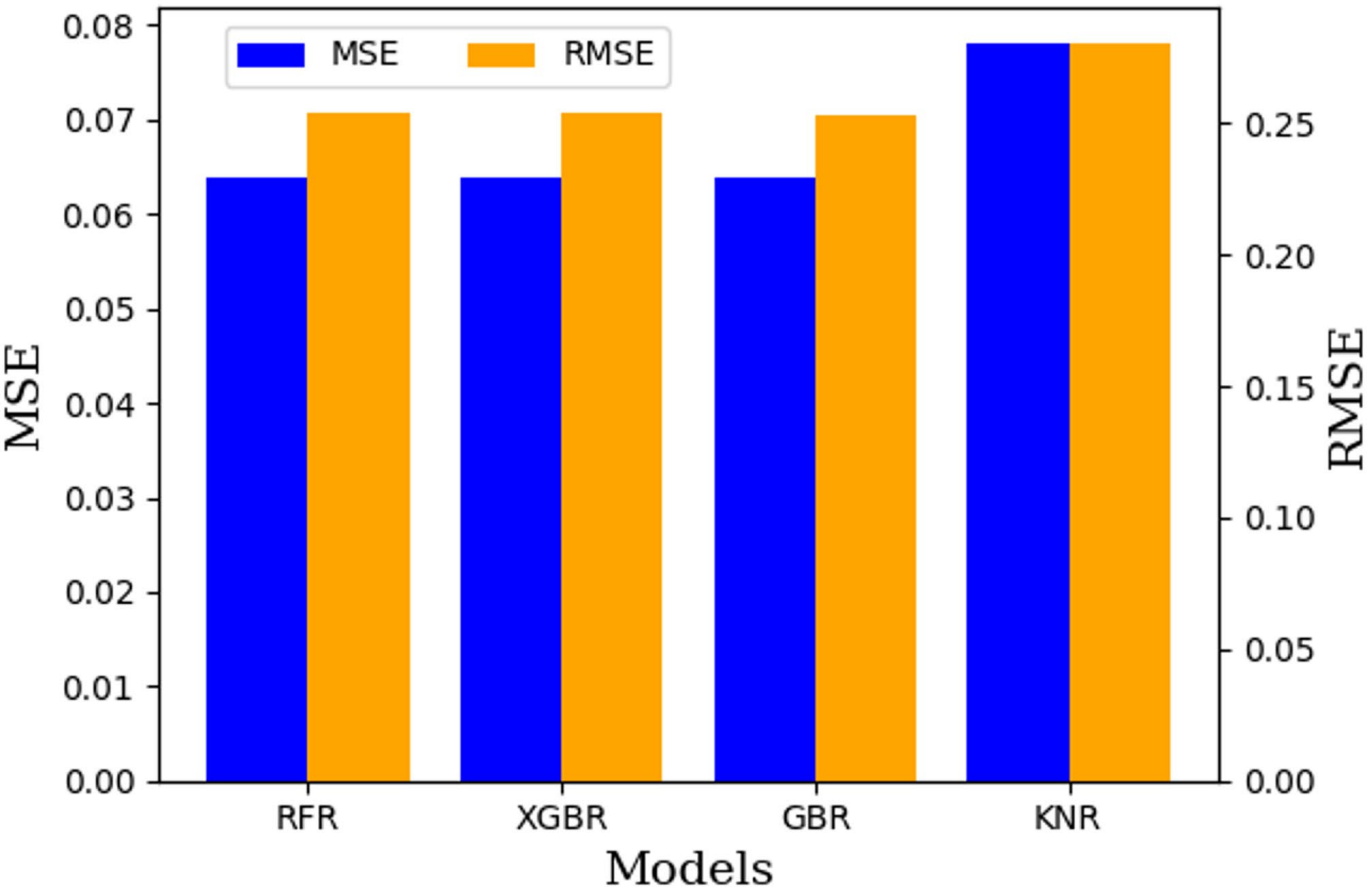
Comparison of the RMSE and MSE values of different machine learning algorithms.

The scatter plots (Figs. 7 and 8), illustrate the relationship between the measured and predicted SN values for each model, offering insights into their accuracy and generalizability. The coefficient of determination (R^2^ values, calculated for the testing dataset, quantify the model fit, with values of 0.916 for RFR, 0.916 for XGBR, 0.917 for GBR, and 0.898 for KNR. The performance evaluation metrics for these models are summarized in Table 4, along with the ranking of the models on the basis of their predictive effectiveness, which helps identify the best-performing model overall. The model with the highest count is considered the best overall model. Figure 9 shows a comparison of the RMSE and MSE values of different machine learning algorithms.

**Fig. 10:**
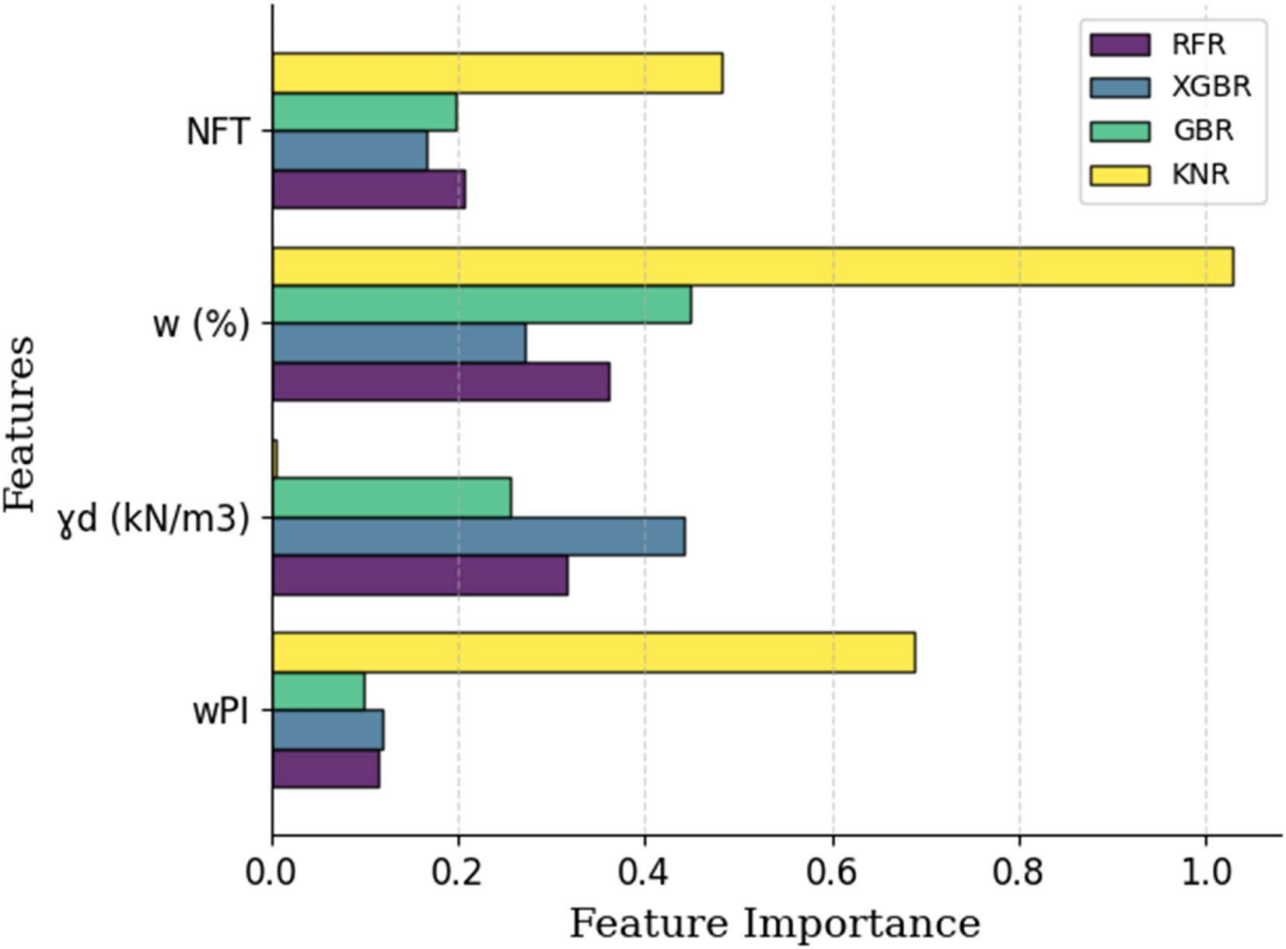
Feature importance of input variables for predicting the SN using machine learning models.

Figure 10 presents the results of the feature importance analysis, which reveals that moisture content is the most influential factor in predicting the SN of flexible pavements in the RFR, XGBR, and KNR machine learning models. In contrast, dry unit weight emerged as the most significant variable in the RFR model. This finding is consistent with the results of^8^who reported that dry unit weight was the most impactful factor on the resilient modulus when using ANN and GEP models. Additionally, Fig. 10 illustrates how feature importance varies across models, reflecting differences in their decision-making processes. While moisture content consistently ranks highest in all models, KNR assigns substantially more weight to both w and wPI compared to the other algorithms, indicating a stronger reliance on these variables.

Although wPI shows a relatively low linear correlation with SN, with a value of 0.12 as displayed in Fig. 1, its feature importance values in Fig. 10 reveal a noticeable impact within the KNR model. This suggests that wPI may influence predictions through nonlinear interactions or localized effects that are more effectively captured by models such as KNR. Unlike tree-based models, KNR does not generalize by applying hierarchical splitting rules. Instead, it relies on the proximity of data points within the feature space. This local distance-based approach allows KNR to detect subtle yet meaningful patterns that may not be reflected by global correlation measures. Such behavior aligns with the characteristics of nearest neighbor algorithms, which are particularly responsive to local structure and variation in the data, as noted by^31^.

For the RFR, XGBR, and KNR models, the dominant influence of moisture content can be explained by well-established geotechnical principles. Moisture content directly affects the strength and stiffness of subgrade soils. An increase in moisture content typically reduces both the soil shear strength and the resilient modulus, which are critical parameters in pavement design. The AASHTO 1993 design equation incorporates the resilient modulus of the subgrade in determining the required structural number for flexible pavements. Increasing moisture content leads to a decrease in the resilient modulus. Since the structural number in this study is directly related to the resilient modulus, it is reasonable that the machine learning models identified moisture content as a dominant predictive factor^13,32,33^.

### Parametric analysis

In this section, a parametric analysis was conducted to evaluate the influence of the AASHTO pavement design parameters such as W₁₈, ZR, S₀, MR, and ΔPSI on the SN. The analysis involved varying each pair of parameters over their plausible design ranges while keeping the remaining parameters fixed at their representative values: W₁₈ = 1 × 10⁶ ESALs, ZR = − 1.282, S₀ = 0.45, MR = 4905.88 psi, and ΔPSI = 2.5. To enhance the robustness and reliability of the solution process, the AASHTO pavement design equation was solved using the bisection method. For each combination of input parameters, SN was computed such that the absolute difference between the left-hand and right-hand sides of the equation did not exceed 0.001.

**Fig. 11:**
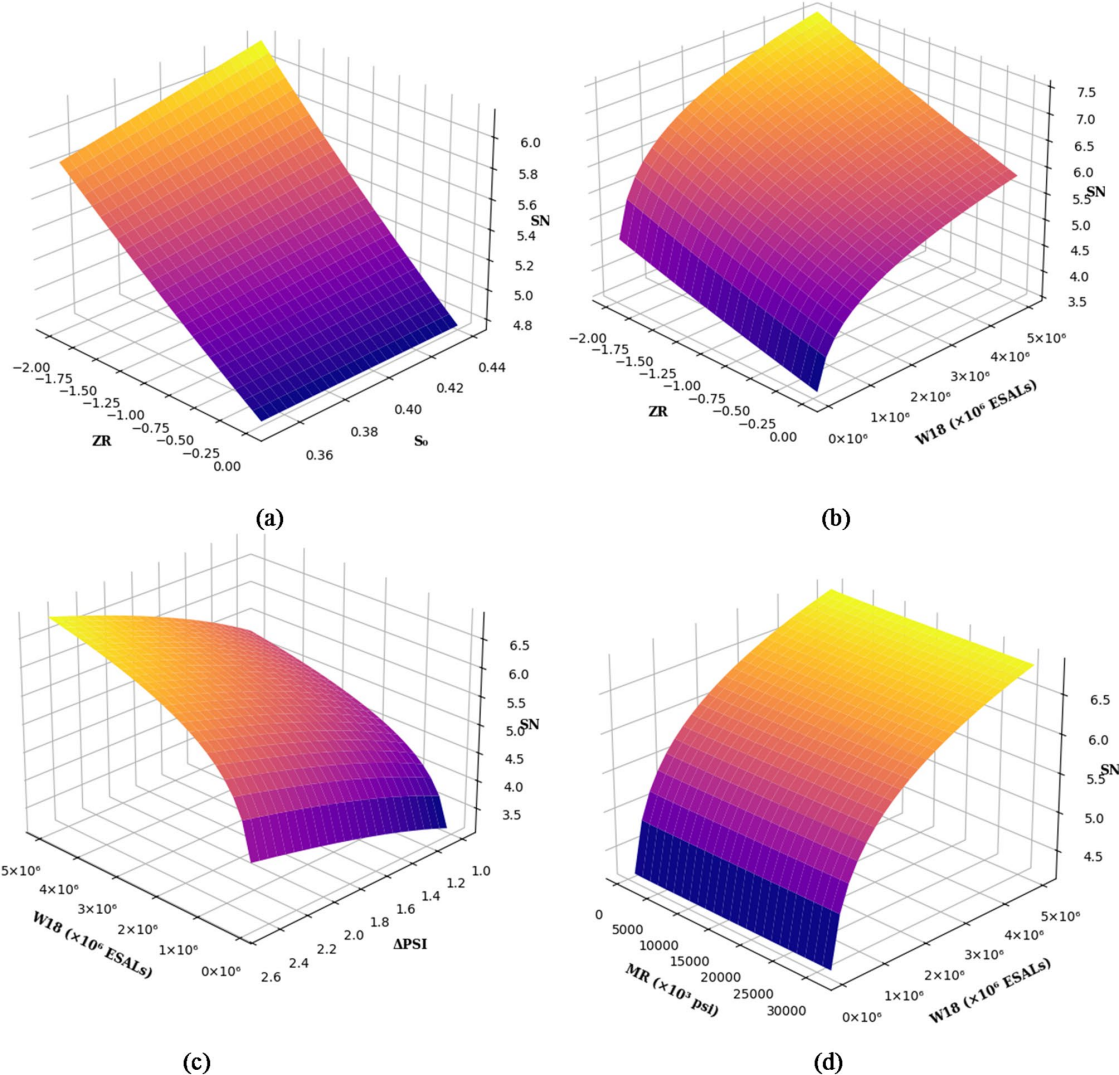
Interactive effects of different input parameters’ combinations on the structural number (SN): (**a**) ZR vs. S₀, (**b**) ZR vs. W18 (×10^4^ ESALs), (**c**) ΔPSI vs. W18 (×10^4^ ESALs) and (**d**) MR (×10^3^ Pa) vs. W18 (×10^4^ ESALs).

In Fig. 11a, the relationship between ZR and S₀ is shown. As reliability improves (ZR becomes more negative), SN increases. Interestingly, when reliability is low (ZR near 0), increasing S₀ yields minimal improvement in SN. However, at higher reliability levels (lower ZR), S₀ has a much stronger impact. Both ZR and S₀ demonstrate linear behavior with SN in this range. Figure 11b displays the interaction between ZR and W₁₈ (expressed in millions of ESALs). SN increases sharply as traffic load rises, and reliability becomes stricter. This relationship is nonlinear, especially at lower traffic values, where small increases in traffic significantly influence SN. In Fig. 11c, the effect of ΔPSI (change in serviceability index) and W₁₈ on SN is shown. As expected, a higher ΔPSI leads to a higher SN, especially under heavier traffic conditions. Figure 11d reveals how MR and W₁₈ affect SN. At lower MR values, SN increases steeply with traffic. As MR rises, this relationship becomes flatter, suggesting diminishing returns in SN improvement. Again, the pattern transitions from nonlinear to nearly linear as traffic exceeds one million ESALs. These observations align with the origins of the AASHTO design method, which was initially developed based on low traffic conditions and weak subgrade materials. As such, the sensitivity of SN to both W₁₈ and MR diminishes under conditions beyond this original scope.

**Table 4:** Comparison of regression model performances based on R^2^RMSE, and MSE. The best model for each metric is highlighted along with the overall model ranking. RMSE and MSE are reported in terms of SN and SN^2^respectively.

Metric	RFR	XGBR	GBR	KNR	Best Model	Overall Ranking
R^2^	0.916	0.916	0.917	0.898	GBR	1
RMSE	0.254	0.254	0.253	0.280	GBR	1
MSE	0.064	0.064	0.064	0.078	RFR / XGBR / GBR	1

## Conclusion

This study aimed to develop and evaluate four machine learning regression algorithms, namely random forest, extreme gradient boosting, gradient boosting, and k nearest neighbors, for predicting the total structural number of flexible pavements using subgrade soil properties and environmental factors. The dataset used in this study, originally compiled by^8^was transformed using the bisection method to estimate structural number values from resilient modulus data.

The results demonstrated that gradient boosting outperformed the other models, achieving the highest coefficient of determination with a value of 0.917 and the lowest values for root mean square error and mean square error, indicating superior predictive accuracy. Random forest and extreme gradient boosting also showed strong performance, particularly in terms of the coefficient of determination, while the k nearest neighbors algorithm produced competitive results.

These findings suggest that the gradient boosting is the most effective model for predicting structural numbers in flexible pavements, offering reliable predictions based on subgrade soil properties and environmental factors. The study highlights the potential of machine learning models in pavement design, particularly in optimizing the design of flexible pavements for different conditions. The integration of machine learning techniques into pavement design could enhance decision making and lead to more efficient, cost-effective infrastructure development.

The feature importance analysis, which was conducted for all the models and is presented in Fig. 10, identified moisture content as the most influential variable, followed by dry unit weight and the number of freeze–thaw cycles.

### Limitations

This study, while offering valuable insights into predicting the structural number (SN) of flexible pavements using machine learning, is subject to certain limitations. The analysis was conducted using fixed AASHTO design parameters, including a specific traffic loading level (W18 = 5 × 10^6^ ESALs), reliability factor (ZR = − 1.282), standard deviation (S_0_ = 0.45), and serviceability loss (ΔPSI = 2.5), which may not fully capture the variability encountered in different roadway scenarios. Additionally, the traffic load was modeled as a constant value, whereas actual ESAL values can vary significantly based on roadway classification and usage. Moreover, due to limited availability of base and subbase data, the model focused on total SN prediction driven primarily by subgrade properties, rather than developing separate layer-specific predictions.

### Recommendations

It is recommended that future researchers extend the current modelling approach by incorporating variable traffic loads. In this study, the prediction was based on a specific traffic load and predefined flexible pavement design parameters, which typically do not vary significantly. However, the traffic load, measured as the number of equivalent single axle load repetitions over the design life of a road, can vary substantially depending on the roadway classification. The incorporation of a broader range of traffic conditions could enhance the model’s applicability across different pavement design scenarios.

Additionally, owing to the limited availability of base and subbase course data, this study focused on predicting the total SN of the pavement, which is influenced primarily by the quality of the subgrade soil. Future studies are encouraged to develop layer-specific models that include the physical and mechanical properties of base and subbase materials. This would enable more detailed and accurate SN predictions for each pavement layer. Such an approach would contribute to a more comprehensive and optimized pavement design framework using machine learning techniques.

## Data Availability

The data that support the findings of this study are available from the corresponding author upon reasonable request.
